# Changes in Serum Protein–Peptide Patterns in Atopic Children Allergic to Plant Storage Proteins

**DOI:** 10.3390/ijms24021804

**Published:** 2023-01-16

**Authors:** Kacper Packi, Joanna Matysiak, Eliza Matuszewska, Anna Bręborowicz, Jan Matysiak

**Affiliations:** 1 Department of Inorganic and Analytical Chemistry, Poznan University of Medical Sciences, 60-780 Poznan, Poland; 2 AllerGen, Center of Personalized Medicine, 97-300 Piotrkow Trybunalski, Poland; 3 Faculty of Health Sciences, Calisia University—Kalisz, 62-800 Kalisz, Poland; 4 Department of Pulmonology, Pediatric Allergy and Clinical Immunology, Poznan University of Medical Sciences, 60-572 Poznan, Poland

**Keywords:** allergy, storage proteins, biomarkers, MALDI-TOF, protein–peptide profiling, anaphylaxis, inflammation, component-resolved diagnostics, personalized medicine

## Abstract

Next to cow’s milk and eggs, plant foods, i.e., legumes, tree nuts and cereal grains, most often sensitise atopic children. Storage proteins constitutes the most relevant protein fraction of plant foods, causing primary sensitisation. They exhibit strong allergenic properties and immunogenicity. Our goal was to analyse sensitisation to 26 plant storage proteins in a group of 76 children aged 0–5 years with chronic symptoms of atopic dermatitis using Allergy Explorer ALEX2 and to discover changes in serum protein–peptide patterns in allergic patients with the use of MALDI-TOF-MS. We reported that 25% of children were allergic to 2S albumins, 19.7% to 7S globulins, 13.2% to 11S globulins and 1.3% to cereal prolamins. The most common allergenic molecules were Ara h 1 (18.4%), Ara h 2 (17.1%), Ara h 6 (15.8%) and Ara h 3 (11.8%) from peanuts, and the mean serum sIgE concentrations in allergic patients were 10.93 kUA/L, 15.353 kUA/L, 15.359 kUA/L and 9.038 kUA/L, respectively. In children allergic to storage proteins compared to the other patients (both allergic and non-allergic), the cell cycle control protein 50A, testis-expressed sequence 13B, DENN domain-containing protein 5A and SKI family transcriptional corepressor 2 were altered. Our results indicate that the IgE-mediated allergy to storage proteins is a huge problem in a group of young, atopic children, and show the potential of proteomic analysis in the prediction of primary sensitisation to plant foods. It is the next crucial step for understanding the molecular consequences of allergy to storage proteins.

## 1. Introduction

Food allergies (FAs) affect over 6% of young children worldwide [1,2]. The strongest and best established risk factor for the development of FA is atopic dermatitis (AD) [3,4,5]. It is reported that 30% of children who develop moderate to severe AD also suffer from FA [6]. The damaged skin barrier of eczema patients is probably a gateway to the absorption of food allergens, contributing to the development of sensitization [7,8]. According to literature data, atopic lesions appear before the onset of food sensitization and FAs most often accompany severe and chronic AD [8].

Next to cow’s milk and eggs, the most common allergens that sensitise atopic children are plant foods: legumes (e.g., peanuts, beans, lentils, chickpeas), tree nuts (e.g., walnut, hazelnut, almond), cereal grains (e.g., wheat, rye, rice, corn, oats), other seeds (e.g., sesame, buckwheat, mustard), vegetables and fruits [9,10]. Food allergens constitute a heterogenic group; among them there are 2S albumins, non-specific lipid transfer proteins (nsLTPs), vicilins (7S globulins), legumins (11S globulins), pathogenesis-related protein family 10 and oleosins [11]. Allergens have diverse pro-inflammatory properties that may increase their allergenic activity, but some of them are still not clearly defined [12]. Certainly, the course and severity of an allergic reaction to individual plant allergens depend on the allergenic properties of a particular protein, its structure, stability, sensitivity to temperature and digestive enzymes [13]. The largest group of defence proteins—apart from proteases and protease inhibitors—are pathogenesis-related proteins (PR), which have enzymatic functions (PR-2: beta-1,3-glucanases), chitinolytic activity (PR 3, PR-4, PR-8, PR-11, chitinases) or regulate the permeability of cell membranes (defensins and lipid transfer proteins) [14]. However, from a clinical point of view, allergens of plant origin that cause the most serious systemic reactions occur in grains and belong to the group (not a family) of storage proteins which participate in the processes of plant germination and growth. Storage proteins are also found in fruit and vegetable seeds (e.g., Act d 13, kiwi) [15]. These allergens are extremely thermostable and resistant to digestive enzymes and bile. The stable structure of storage proteins results from the presence of numerous disulphide bridges [16]. Heating and other food processing methods have little effect on the allergenic activity of storage proteins [17]. It has been shown that roasting peanuts increased their allergenicity, i.e., the IgE binding capacity and stability of the allergens, due to the formation of stable molecular aggregates [18]. Because of their stability, storage proteins are digested only partially in the stomach, and therefore, immunologically active allergens can reach the small intestine and the circulation [17,19,20]. Patients allergic to storage proteins from legumes, nuts or seeds may thus react with severe systemic symptoms and even life-threatening anaphylaxis [11]. The group of storage proteins includes structural proteins from the cupin superfamily (7S globulins—vicilins and 11S globulins—legumins) and some proteins from the prolamin superfamily (2S albumins and cereal prolamins) [21,22]. Storage proteins are most often the major allergens that are the cause of primary sensitization. At the same time, storage proteins give unexpected cross-reactions, even in spite of a small homology between them, and therefore, the knowledge of the mutual relationships of allergens belonging to this group is the basis for the correct diagnosis of many serious allergies and the assessment of the risk of anaphylactic reaction [23]. To date, a growing number of allergenic molecules and their isoforms have been discovered, purified, sequenced and cloned [24]. It allows studying the immunologic reactivity and the degree of cross-reactivity of these allergen proteins, which leads to the creation of new diagnostic methods/algorithms for clinicians.

The aims of this study were as follows:

(1) To analyse sensitisation to plant storage proteins in a group of children aged 0–5 years with chronic symptoms of atopic dermatitis.
(2) To discover changes in serum protein–peptide patterns in atopic children allergic to plant storage proteins.

We evaluated the detailed profile of sensitisation to 26 plant storage proteins in a Polish population of young children aged 0–5 years with chronic symptoms of atopic dermatitis. Molecular ALEX2 Allergy Explorer (MacroArray Diagnostics, Wien, Austria) was applied. Next, we investigated the influence of IgE-mediated allergy to plant storage proteins on the patients’ proteomic profile using the MALDI-TOF (matrix-assisted laser desorption/ionization time of flight) mass spectrometer. To our knowledge, there has been no research focusing on the proteomic profiling in children allergic to plant storage proteins. We would like to emphasize the essence of the problem of primary allergy to plant foods (legumes, tree nuts, cereal grains, other seeds, fruits) in a group of young, atopic children. We also would like to discover changes in protein expression in these patients and to point out proteins and peptides involved in allergic inflammation. The results may contribute to a better understanding of the mechanism and molecular consequences of primary allergy to storage proteins. A compilation of proteomic data can explain the molecular pathogenesis of storage protein allergy and provide the basis for better diagnosis and appropriate personalized treatment.

## 2. Results

### 2.1. Allergy to Plant Storage Proteins—Sensitisation Patterns in Atopic Children

Using the ALEX2 test, we measured the serum concentration of IgE specific to 26 plant storage proteins: Ara h 1, Cor a 11, Jug r 6, Jug r 2, Pis v 3, Gly m 5, Ara h 3, Cor a 9, Jug r 4, Gly m 6, Ana o 2, Pis v 2, Ara h 2, Ara h 6, Cor a 14, Ses i 1, Sin a 1, Jug r 1, Ana o 3, Mac i, Ber e 1, Pis v 1, Pap s, Fag e 2, Gly m 8 and Tri a 19. Among all studied proteins, 6 belonged to the family of vicilins (7S globulins), 6 to legumins (11S globulins), 13 to 2S albumins and 1 allergen to the family of cereal prolamins.

**Figure 1:**
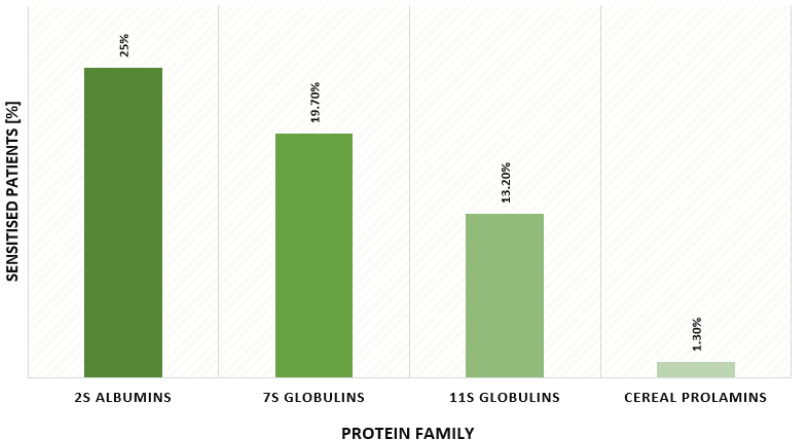
Prevalence of sensitisation to 2S albumins, 7S globulins, 11S globulins and cereal prolamins in atopic children aged 0–5 years.

In a group of 76 atopic children aged 0 to 5 years, we detected a higher concentration of sIgE (<0.30 kUA/L) against plant storage proteins in the serum of 22 individuals. All of these patients were sensitised to at least one storage protein and exhibited the full symptoms of primary allergy to plant foods. Moreover, 25% of atopic children were allergic to 2S albumin, 19.7% to 7S globulins, 13.2% to 11S globulins and 1.3% to the family of cereal prolamins (Figure 1). The complete results are presented in Table 1.

**Figure 2:**
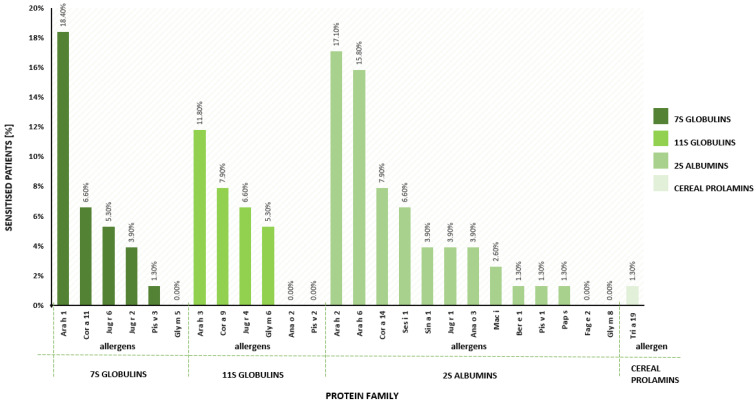
Prevalence of sensitisation to 26 allergenic molecules in atopic children aged 0–5 years.

**Figure 3:**
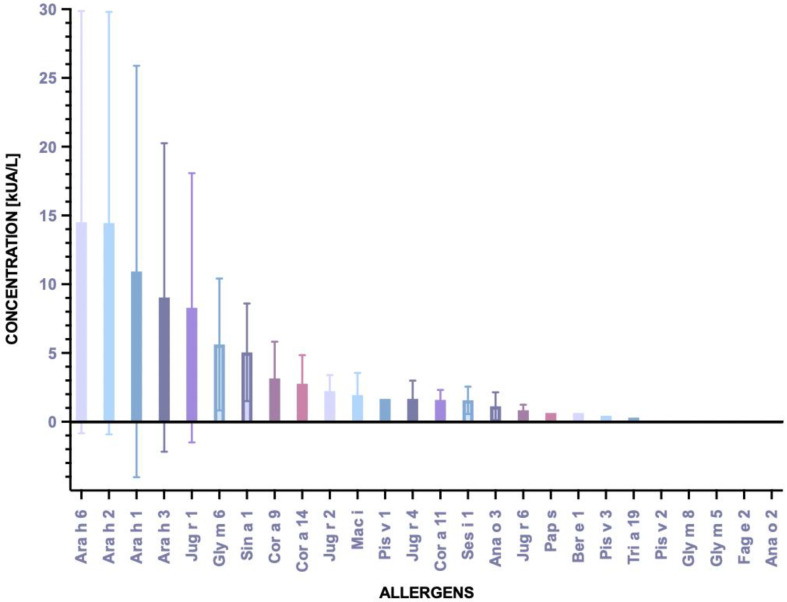
Values of sIgE concentration for 26 storage proteins.

The most common allergens from the family of vicilin, legumin and 2S albumin in the population of children aged 0–5 years with chronic symptoms of atopic dermatitis were peanuts: Ara h 1 (18.4%), Ara h 2 (17.1%), Ara h 6 (15.8%) and Ara h 3 (11.8%) (Figure 2). In the allergy group, the highest mean concentration of IgE was detected for Ara h 6 (15.359 kUA/L) and Ara h 2 (15.353 kUA/L) (Figure 3).

### 2.2. Proteomic Features Characterization of Allergy to Plant Storage Proteins

#### 2.2.1. Protein–Peptide Profiling

**Figure 4:**
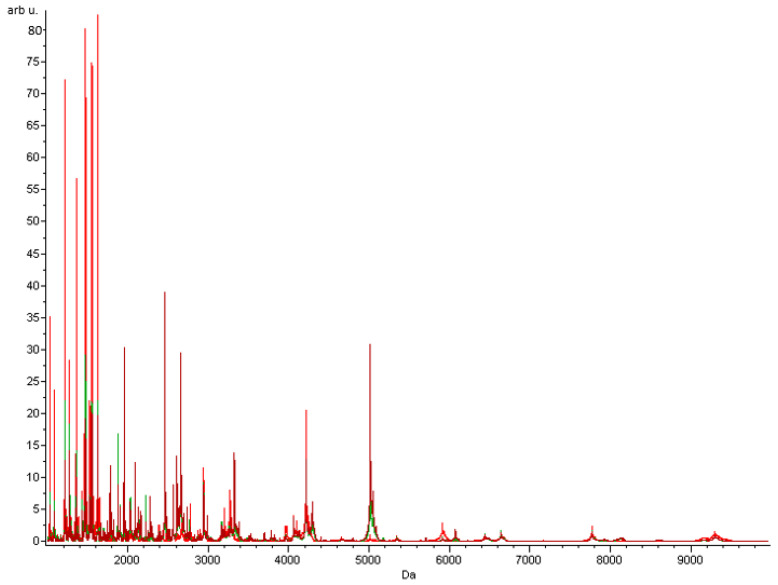
Average MALDI-TOF MS spectra of serum samples characteristic of study groups. Spectra of patients allergic to plant storage proteins (red) and controls (green) are presented over the full scan range of *m*/*z* 1000–10,000.

Seventy-six serum samples from atopic children allergic to plant storage proteins (*n* = 22) and controls (*n* = 54) were pre-treated with ZipTips and analysed in triplicate by MALDI-TOF MS. The average MALDI-TOF MS spectra of the study groups are shown in Figure 4. Three mathematical algorithms were used to analyse the obtained data: genetic algorithm (GA), supervised neural network (SNN) and quick classifier (QC). All these algorithms differ in methodology; hence, the peaks defined as discriminating for each of them are different (Table 2). However, peaks (*m*/*z* 3689.35 and 4964.58) were present in both the genetic algorithm and the quick classifier, while another peak (*m*/*z* 1795.39) was common to the quick classifier and the supervised neural network. The recognition capability and cross-validation were calculated for all algorithms (Table 3). The highest value of average cross-validation (94.91%) and the recognition capability (99.25%) from three replicates was obtained using the genetic algorithm. An assessment of the discrimination ability of each peak was obtained by calculating the receiver operating characteristic (ROC) curve and the area under the ROC curve (AUC). In the mass range of *m*/*z* 1000–10,000, the highest AUC value (0.68) was calculated for a peptide of *m*/*z* 2144.89, classified as discriminative by the quick classifier algorithm.

#### 2.2.2. Identification of the Discriminatory Features

To identify the peaks which statistically had the highest diagnostic efficacy, serum samples were pre-treated with ZipTips and examined by nanoLC-MALDI-TOF/TOF-MS/MS tandem mass spectrometry. We identified five proteins, i.e., cell cycle control protein 50A, testis-expressed sequence 13B, DENN domain-containing protein 5A, SKI family transcriptional corepressor 2 and sperm-associated antigen 6, using the SwissProt database and Mascot search engine. However, according to the statistics (the Wilcoxon; *p*-value ≤ 0.05), the four detected features fully differentiate patients with storage protein allergy from the control group (Table 4).

Fragmentation of signal *m*/*z* 1077.88 allowed us to identify the peptide sequence R.NSSNTADITI.- with a high score in the Mascot database assigned to cell cycle control protein 50A (CC50A_HUMAN). Testis-expressed sequence 13B (TX13B_HUMAN) and DENN domain-containing protein 5A (DEN5A_HUMAN) were other identified proteins corresponding to peaks of *m*/*z* 1419.92 and 2012.59, respectively, with the peptide sequences K.EACTWGSLALGVR.F and K.LNTGQIQESIGEAVNGIVK.H. The MS/MS analysis of precursor ion *m*/*z* 2601.66 resulted in the identification of SKI family transcriptional corepressor 2 based on the peptide sequence K.AGGGSYHHSSAFRPVGGKDDAESLAK.L with a significant hit in the Mascot search. The spectra were analysed in the reflector mode in the mass range of 700–3500 Da. For this reason, discriminative peaks with mass of *m*/*z* 3689.35, 4151.16, 4287.68, 4396.62, 4645.8, 4964.58, 5004.83, 5017.61, 5044.33, 5352.47, 5905.01, 6065.66, 6221.79, 7922.04, 8126.66, 8601.73, 8866.89 and 9135.64 were not identified. The identification of peaks *m*/*z* 1219.27, 1277.39, 1450.43, 1773.08, 1795.39, 1897.85, 1945.54, 2144.89, 2269.28, 2474.89, 2726.71, 3159.56 and 3198.01 also requires further analysis.

## 3. Discussion

### 3.1. Allergy to Plant Storage Proteins-Sensitisation Pattern in Atopic Children

2S albumins, 7S globulins, 11S globulins and cereal prolamins belong to the group of storage proteins. They are the most clinically significant plant proteins, responsible for primary allergies to legumes, nuts and seeds, often causing severe reactions. In a large multicentre study including both children and adults, IgE specific to storage proteins was found only in patients who developed allergy before the age of 14 [25]. We examined the sensitisation to 26 plant storage proteins in a Polish population of atopic children aged 0–5 years using Allergy Explorer ALEX2. Macroarray nanotechnology-based immunoassay ALEX2 is commercialized as quantitative in nature, and its specificity is improved by inhibiting the reactivity of cross-reactive carbohydrate determinants (CCD) [26]. Studies show a substantial agreement between the multiplex macroarray ALEX2 and the singleplex test ImmunoCAP [26]. According to our results, 25% of children with chronic symptoms of atopic dermatitis were allergic to 2S albumins, 19.7% to 7S globulins, 13.2% to 11S globulins and 1.3% to the family of cereal prolamins.

2S albumins belong to the prolamin superfamily. They are characterized by a high content of proline and glutamine amino acids, and their structure is rich in alpha helices and disulphide bridges connecting them, which ensures extraordinary durability in relation to temperature and digestive enzymes. 2S albumins are mainly found in grains, and their main role is to provide nitrogen and sulphur during the germination of the plant. They are class I food allergens—primarily sensitising, and due to their stable structure, 2S albumins can cause systemic reactions. 2S albumin allergens cause the most severe and common anaphylactic reactions of all allergens [27]. According to the literature, the frequency of sensitization to 2S albumin allergens among Polish atopic children aged 0–18 years is high and amounts to 38%. 2S albumins were the cause of anaphylaxis in 32% of the 237 anaphylactic children [23]. This is consistent with our results. In the group of 76 children aged 0–5 years with chronic symptoms of atopic dermatitis, allergy to 2S albumin was the most common and amounted to 25%. In the 2S albumin family, Polish children are most often allergic to the allergens Ara h 2 (59%) and Ara h 6 (50%) of peanut and Ses i 2 of sesame (51%) [23]. Specific IgE against Ara h 2 and Ara h 6 was present in 76–96% of children suffering from peanut allergy in the USA and Central and Northern Europe [28]. The most clinically significant allergen among the 2S albumins is Ara h 2, one of the major peanut allergens [24,29]. In peanut allergy, approximately 90% of patients sensitised to Ara h 2 suffer from a severe peanut allergy, while only 70% of patients with IgE to peanut extract are truly allergic [11,30]. IgE to Ara h 2 ≥ 5 kU/L can classify Dutch children as peanut allergic, while the absence of IgE to Ara h 2 can be used to rule out class I peanut allergy [31]. In our study group, over 17% of individuals were sensitised to Ara h 2 and they were truly allergic to peanut. Cross-reactions are possible both within 2S albumin (Ana o 3 cashew and Pis v 1 pistachio; Act d 13 kiwi and Ara h 2 peanut; Jug r 1 walnut and Car i 1 pecan) and, less frequently, within the entire group of storage proteins (Ara h 2 from Ara h 1 and Ara h 3 of peanut) [32,33,34]. Clinical cross-reactions between the 2S albumin Ara h 2 and Cor a 14 of hazelnut and between Ara h 2 and Ana o 3 of cashew are rare [35]. Sensitization to 2S cashew albumin (Ana o 3) increases the risk of a severe anaphylactic reaction 15-fold. The endotype most at risk of severe anaphylaxis is monovalent sensitisation to Ana o 3, without sensitisation to other components of food allergens [27]. In three participants in the study group, we detected allergy to Ana o 3. One patient experienced anaphylactic shock, but it was not a monovalent sensitization to the cashew allergen Ana o 3. In a German multicentre study, all cashew-allergic children were sensitised to Ana o 3 [36]. No patient without Ana o 3-sensitization was allergic. In receiver operation curves, Ana o 3 discriminated between allergic and tolerant children with an area under the curve of 0.94 [36]. IgE to cashew nut Ana o 3 is highly predictive for cashew nut allergy and discriminated between allergic and tolerant children better than cashew nut extract-specific IgE [11,34].

7S globulins (vicilins) are trimers with a molecular weight of 150–190 kDa composed of three homogeneous polypeptide chains. These proteins fold to create stable pairs of barrel-like structures formed from β-sheets. They are resistant to temperature and digestion, and they can cause an anaphylactic reaction. According to literature data, the frequency of sensitisation to 7S globulin allergens among Polish atopic children aged 0–18 is high—34% [23]. In our group of children aged 0–5 years, the incidence of sensitisation to 7S globulin was 19.7%. The most clinically significant allergen among the 7S globulins is Ara h 1 of peanut. In allergic patients, the concentration of IgE specific to Ara h 1 was high and amounted to 14.96 kUA/L. In the Polish population, sensitisation to the walnut allergen Jug r 2 is quite common. However, isolated sensitisation to nJug r 2 (natural purified allergen) in many cases is due to the presence of antibodies against carbohydrate determinants (CCD), because this allergen is glycosylated [37]. We did not report monovalent sensitisation to nJug r 2. Population-based studies concerning nut allergy prevalence for Central Europe in adults showed very few sensitisations (between 0 and 0.4%) for walnut Jug r 2 [10]. 7S globulins are important allergens in infants and preschool children (especially Ara h 1 sensitisation), but systemic reactions are rare. In our study, 18.4% of participants were allergic to Ara h 1, of which only one case was a monovalent sensitisation to 7S globulins. This patient had no systemic reaction after peanut exposure. Cross-reactions with other storage proteins are rare and occur mainly within the 7S globulins, e.g., Pis v 3 (pistachio) and Ana o 1 (cashew), and Jug r 2 (walnut) and Car i 2 (pecan) [34,38,39]. On the other hand, despite the lack of structural similarity, cross-reactions between the peanut storage proteins Ara h 1, Ara h 2 and Ara h 3 are observed [28,29,40]. Thus, in our study, as many as 9 out of 17 patients allergic to peanut had an increased concentration of IgE antibodies specific to Ara h 1, Ara h 2 and Ara h 3. It was shown that the cross-reactive IgE antibodies bind to unstructured loops, which are exposed at the allergen’s surface and whose sequences exhibit similarities between these unrelated proteins [40,41]. This observation also explains why the majority of peanut allergic individuals showed sensitization to all three major allergens (Table 5).

11S globulins (legumins) are hexamers composed of three double polypeptide chains connected by disulphide bridges. They belong to storage proteins, so they are thermostable and resistant to heat and can cause an anaphylactic reaction. The frequency of sensitisation to 11S globulin allergens among Polish atopic children aged 0–18 is 25%. In children aged 0–5 with chronic symptoms of atopic dermatitis, we obtained 13.2%. The most clinically significant allergen among 11S globulins in Poland is the main hazelnut protein Cor a 9 [23]. Sensitisation to Cor a 9 increases the risk of severe anaphylactic reaction by more than 6-fold [27,42]. In our group, three of six patients allergic to Cor a 9 had a systemic reaction in the form of dyspnoea and angioedema. The parents of the patients were able to relate the allergic reaction to the consumption of products containing hazelnuts. According to population-based study conducted by Burney et al., the highest sensitisation rate was shown against Cor a 9 in Sofia, with 3%, and the lowest was 0% in Utrecht [10]. Cross-reactions among 11S globulins occur rarely, but much more often than in the case of other storage proteins, i.e., 2S albumins or 7S globulins [43,44]. The best-known cross-reactions are Sin a 2 (mustard) and Ara h 3 (peanut); Act d 12 (kiwi seeds) and Ara h 3; Fag e 1 (buckwheat) and Ses i 6 (sesame); Pap s 11S (poppy seed) and Cor a 9 (hazelnut); Ana o 2 (cashew nut) and Pis v 5 (pistachio); and Jug r 4 (walnut) and Car i 4 (pecan) [44,45]. Linear IgE epitopes of Ara h 3, Cor a 9, Jug r 4 and Ana o 2 were observed to be exposed to the surface and to exhibit similar conformations [46].

The biomarker for cereal prolamins is Tria 19 (gliadin), the main wheat allergen, which has mostly linear epitopes resistant to temperature and digestion [47]. Tria 19 is often responsible for food-dependent exercise-induced anaphylaxis (FDEIA). According to the literature, the frequency of sensitisation to cereal prolamin allergens among Polish atopic children is low and amounts to 3.4%. However, the clinical significance of cereal prolamins is extremely important because they cause severe systemic reactions, also in infants [11,23]. In our study group, the child allergic to Tria 19 was 14 months old.

IgE specific to seed storage proteins generally has a high predictive value in diagnosing food allergies. The measurement of IgE specific to seed storage proteins is a useful tool in the diagnostic process of peanut, tree nut and seed allergy and it has a higher diagnostic value than measurement of IgE to whole allergen extracts [11]. However, the problem is that not all relevant allergenic seed storage proteins are available for routine diagnosis. Moreover, the clinical relevance of IgE co-sensitisation and the effects of cross-reactivity are largely unknown and still need to be investigated. The obtained results indicated that the IgE-mediated allergy to storage proteins is a huge problem in a group of atopic children aged 0–5 years. Despite the small number of participants, our results are in line with the literature data. The analysis of the sensitisation profile introduces the study of changes in protein–peptide patterns in our patients.

### 3.2. Proteomic Features Characterization of Allergy to Plant Storage Proteins

In Europe and in North America, most primary allergies to nuts, legumes and seeds involving systemic, severe reactions are caused by storage proteins. We discussed earlier that storage proteins exhibit strong allergenic properties and immunogenicity. In this study, apart from the analysis of sensitisation profiles, we aimed to discover changes in serum protein–peptide patterns in atopic children allergic to plant storage proteins. As we previously reported, analysis of serum protein–peptide patterns derived from allergic patients revealed changes in expressed proteins involving fibrinogen alpha chain, coagulation factor XIII chain A, complement C4-A and inter-alpha-trypsin inhibitor heavy chain H4 [48]. On the other hand, Pavel et al. demonstrated changes in the skin proteome of patients suffer from atopic dermatitis [49]. They showed significant upregulation in inflammatory markers, i.e., matrix metalloproteinase 12, Th1/C-X-C motif chemokine 10, Th17/Th22/PI3, CCL20 and S100A12, and in cardiovascular-associated proteins, i.e., matrix metalloproteinases, E-selectin, platelet growth factor, fatty acid binding protein 4, myeloperoxidase and vascular endothelial growth factor A. In atopic children who suffer from primary allergy to plant foods compared to the other patients (allergic and non-allergic), the cell cycle control protein 50A, testis-expressed sequence 13B, DENN domain-containing protein 5A and SKI family transcriptional corepressor 2 were altered.

In the present study, we identified cell cycle control protein 50A (CDC50A) as a feature differentiating patients allergic to plant storage proteins and the control group. Fragment of this protein was identified for the peak of *m*/*z* 1077.88 classified as discriminative in genetic algorithm. CDC50A (alternative names: P4-ATPase flippase complex beta subunit TMEM30A, transmembrane protein 30A) belongs to the CDC50 family of membrane proteins, which contain two transmembrane segments with short N- and C-cytoplasmic regions and a large extracellular loop [50,51]. It is a β-subunit of the fippase complex. CDC50 combined with P4-ATPase catalyses the hydrolysis of ATP coupled to the transport of aminophospholipids from the outer to the inner leaflet of various membranes and ensures the maintenance of asymmetric distribution of phospholipids [52]. The biological function of cell cycle control protein 50A is largely unexplored. It has been reported that CDC50A may be associated with the process of angiogenesis [53] and may also play an important role in cell migration. According to literature data, overexpression of CDC50A induced extensive cell spreading and markedly increased cell migration [54]. CDC50A has also been reported to play a critical role in the survival of hematopoietic stem cells [55]. CDC50A has previously been detected in immune cells; however, we discovered for the first time that CDC50A may be altered in a severe allergic reaction related to plant storage proteins.

**Figure 5:**
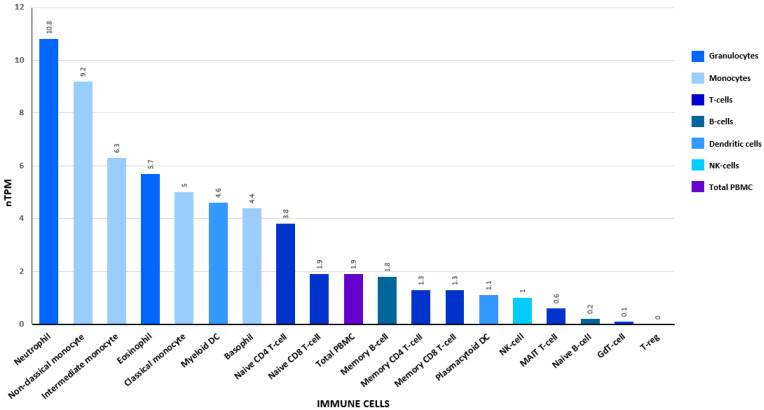
Transcript expression values calculated as nTPM (transcript per million) for 18 immune cell types and total peripheral blood mononuclear cells based on the Human Protein Atlas (HPA) dataset (https://www.proteinatlas.org/ (accessed on 7 December 2022)).

DENN domain-containing protein 5A (DENND5A), another protein identified in this study, acts as a RAB-activating guanine nucleotide exchange factor (GEF) [56]. The main function of DENND5A is to catalyse the conversion of GDP to GTP. It changes inactive GDP-bound Rab proteins to their active GTP-bound form. DENN domain-containing protein 5A is recruited by RAB6/RAB39 onto the Golgi apparatus [57]. There are reports that mutations in the gene encoding the DENND5A protein are associated with early childhood epileptic encephalopathy [58]. To date, DENN domain-containing protein 5A has been detected in all immune cell lineage (Figure 5). Based on the Human Protein Atlas (HPA) dataset, DENND5A transcript expression in neutrophils, monocytes, eosinophils, basophils and naive CD4 T-cells were higher than in total peripheral blood mononuclear cells.

Precursor ion *m*/*z* 1419.92 has been identified as part of SKI family transcriptional corepressor 2 (SKOR2). SKOR2 is a protein of approximately 30 kDa and 297 amino acids, characterized by a SKI homologue domain and a SAND domain [59]. It is expressed both inside and outside of the nucleus [60]. SKOR2 exhibits transcriptional repressor activity. This protein inhibits transforming growth factor β (TGF-β), which regulates cell growth [59]. SKOR2 is mainly expressed in the cerebellum, spinal cord and testis. According to the literature data, in the physiological state, SKOR2 is present in cells at low levels, but overexpression of this protein is characteristic of tumour cells [61].

In this study, testis-expressed sequence 13B (TX13B) was the last identified feature differentiating the studied groups. TX13B is about 35 kDa and 312 amino acids in length. The molecular properties and biological function of this protein have not yet been determined. There are two conserved sequence motifs: FIN and LAL [62,63]. TX13B interacts with two proteins, hippocalcin-like protein 4 and visinin-like protein 1, which are involved in the calcium-dependent regulation of rhodopsin phosphorylation [64].

**Figure 6:**
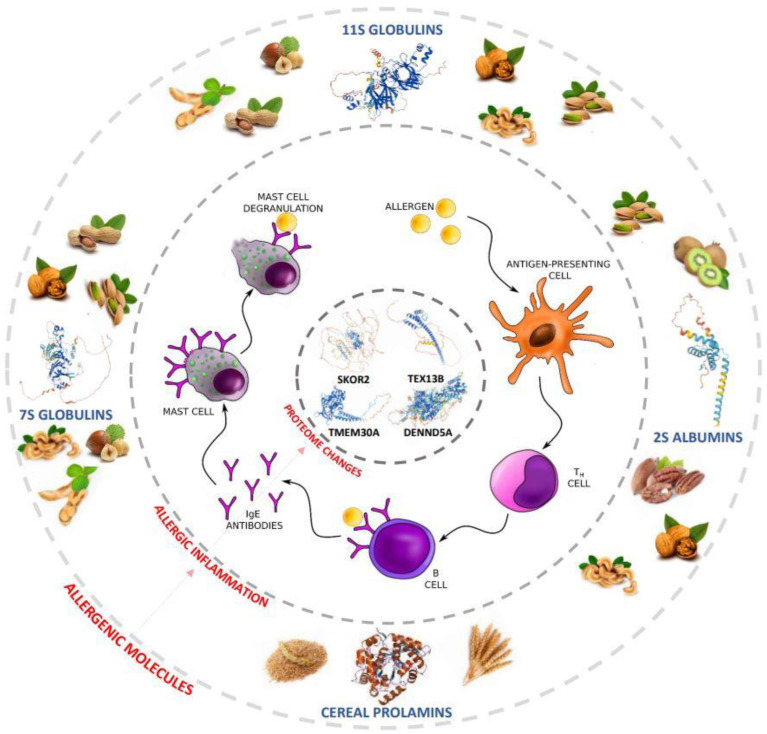
A cascade of molecular changes driven by plant storage proteins. SKOR2-SKI family transcriptional corepressor 2; TEX13B-testis-expressed sequence 13B; TMEM30A-P4-ATPase flippase complex beta subunit TMEM30A/transmembrane protein 30A/cell cycle control protein 50A (CDC50A); DENND5A-DENN domain-containing protein 5A.

These results confirm that primary plant allergy triggers biochemical processes, as exemplified by measurable modifications in the expressed proteome (Figure 6). All of the identified proteins may be associated with the inflammatory response in a course of allergy to storage proteins. However, many functions of the mentioned proteins are still unknown. This is only the first study aimed to discover and identify peptides with discriminatory power between patients allergic to storage proteins and other allergy suffers and healthy individuals. It is a crucial step for understanding the mechanism of pathological processes and the molecular consequences of allergy to storage proteins. Peaks of *m*/*z* 1219.27, 1277.39, 1450.43, 1773.08, 1795.39, 1897.85, 1945.54, 2144.89, 2269.28, 2474.89, 2726.71, 3159.56 and 3198.01 have remained unidentified and require further study. The possible reason is the presence of adjacent peaks or reduced fragmentation of the peptide [28]. Intact peptides detected in linear mode and statistically classified as discriminating between study groups must be identified in their undigested form.

Nowadays, the greatest potential for component-resolved diagnostics lies in distinguishing primary food allergies from pollen-associated food allergies, especially in case of legumes, tree nuts, cereal grains vegetables and fruits [11]. So far, the clinical significance of serological cross-reactivity between different seeds, legumes and nuts has not been deduced with certainty, even on the basis of molecular allergy diagnostics [30]. New diagnostic tools may be offered by proteomics, which is one of the most promising strategies for searching for potential biomarkers and assessing differences between people with different health statuses [65]. The results of this study indicate the potential of proteomic analysis in the prediction of primary allergy to plant foods. Visible changes in the proteome may contribute to the early diagnosis of allergy, the assessment of the severity of an allergic reaction, the prediction of anaphylaxis and, most importantly, they can act as markers for primary genuine IgE sensitisation.

## 4. Materials and Methods

### 4.1. Study Group and Sample Collection

The study was approved by the Bioethical Commission of Poznan University of Medical Sciences. The parents of participants signed informed consent. They were given an explanation of the main objectives and possible benefits of the study.

A total of 76 children aged 0 to 5 years with chronic symptoms of AD (L20) participated in the study. Some patients developed acute symptoms, i.e., urticaria (L50), angioedema (T78.3) and/or anaphylaxis (T78.0) following food exposure. The parents of the patients carefully completed the questionnaire, and then all participants underwent a detailed medical examination. The demographic profiles of participants are shown in Table 6.

Venous blood samples obtained from study participants were incubated and centrifuged. Collected sera were stored in −80 °C until analysis.

### 4.2. Measurement of sIgE Serum Levels

We examined sensitisation to 26 plant storage proteins in the Polish population of children aged 0–5 years with chronic symptoms of AD using Allergy Explorer ALEX2 test (MacroArray Diagnostics, Wien, Austria). We investigated the concentration of IgE specific to Ara h 1, Cor a 11, Jug r 6, Jug r 2, Pis v 3, Gly m 5, Ara h 3, Cor a 9, Jug r 4, Gly m 6, Ana o 2, Pis v 2, Ara h 2, Ara h 6, Cor a 14, Ses i 1, Sin a 1, Jug r 1, Ana o 3, Mac i, Ber e 1, Pis v 1, Pap s, Fag e 2, Gly m 8 and Tri a 19.

Allergy Explorer2 (ALEX2) is an in vitro quantitative diagnostic test for the determination of allergen-specific IgE (sIgE). Allergen extracts or molecular allergens, which are conjugated with nanoparticles, are deposited systematically on the solid phase, forming a macroscopic array. First, the cartridge chip is incubated with 0.5 mL of a 1:5 dilution of serum with agitation. During this step, immobilized allergens react with specific IgE present in the patient’s sample. The serum diluent contains a CCD inhibitor, which guarantees 85% CCD inhibition. After incubation for two hours, non-specific IgE is washed away. Anti-human IgE labelled with alkaline phosphatase is then added and incubated for 30 min. After a second washing step, substrate is added. Finally, after eight minutes the reaction is stopped by adding a blocking reagent. The membranes are dried, and the intensity of the colour reaction for each allergen spot is measured. The results were analysed using MADx’s Raptor Analysis Software and reported in IgE response units (kUA/L). We considered the concentration ≥0.35 kUA/L to be positive [26].

Based on the results of the Allergy Explorer ALEX 2 test, medical history and oral food challenge tests (OFC), the participants were divided into target groups. The study group contained 22 young children suffering from AD and diagnosed with IgE-mediated allergy to at least one plant storage protein belonging to the family of viscilins, legumins, 2S albumins or cereal prolamins. All patients sensitised to storage proteins exhibited the full symptoms of primary allergy to plant foods. The control group consisted of 54 atopic patients without IgE-mediated allergy to storage proteins (27 patients without IgE-mediated food and without inhalant allergy; 13 patients with only inhalant allergy; 6 patients with only IgE-mediated food allergy other than plant storage proteins; 8 patients with both inhalation and IgE-mediated food allergy other than plant storage proteins). The sera of all patients were subjected to MALDI-TOF examination to characterize the proteomic profile of patients suffering from allergy to proteins contained in plant foods.

### 4.3. Pre-Treatment of the Serum Samples

In order to desalt and concentrate the samples, prior to the MALDI-TOF MS (matrix-assisted laser desorption/ionization time of flight mass spectrometry) analysis, the solid phase extraction method based on ZipTip C18 pipette tips was used in accordance with the manufacturer’s instructions (Millipore, Bedford, MA, USA). First, serum samples were diluted in 0.1% trifluoroacetic acid (TFA) in water (1:5 ratio). The tips were conditioned with acetonitrile (ACN) and 0.1% TFA. The prepared samples were then loaded onto the ZipTip pipette tips. After washing in 0.1% TFA, the bound peptides were eluted with 4 μL of 50% ACN in 0.1% TFA.

### 4.4. MALDI-TOF-MS Protein and Peptide Profiling

After ZipTip purification and pre-concentration of samples, MALDI-TOF MS analysis was performed as previously described [66]. Briefly, 1 μL of each eluent sample was mixed with 10 μL of 0.3 g/L a-cyano-4-hydroxycinnamic acid (HCCA) matrix solution in ethanol:acetone (2:1 ratio, *v*/*v*) and then the mixture was spotted onto AnchorChip Standard 800 μm (Bruker Daltonics, Bremen, Germany) target plate in triplicate and allowed to crystallize at room temperature. A MALDI-TOF/TOF UltrafleXtreme (Bruker Daltonics, Bremen, Germany) mass spectrometer was used to perform MS analyses in the linear positive mode. The spectra were acquired from an average of 2000 laser shots per sample in the *m*/*z* range of 1000–10,000. The MS spectra were externally calibrated with the mixture of Protein Calibration Standard I and Peptide Calibration Standard (BrukerDaltonics, Bremen, Germany) (5:1, *v*/*v*). The average mass deviation from reference masses was less than 100 ppm. The MS parameters for the analysis were as follows: ion source 1, 25.09 kV; ion source 2, 23.80 kV; matrix suppression mass cut-off *m*/*z*, 700 Da; pulsed ion extraction, 260 ns; lens, 6.40 kV. FlexControl 3.4 software (Bruker Daltonics, Bremen, Germany) was applied for the acquisition and processing of MS spectra. Inter-day and intra-day reproducibility of the applied protocol were assessed previously [67]. The samples were analysed in a random order.

### 4.5. NanoLC-MALDI-TOF/TOF MS Identification of Discriminative Peaks

Identification of the discriminative peptides between the studied groups was performer using a nano-liquid chromatography–matrix-assisted laser desorption/ionisation–time-of-flight/time-of-flight mass spectrometry (nanoLC-MALDI-TOF/TOF MS) system. The sample was prepared with the ZipTip technique. The obtained eluent was further subjected to nanoLC separation. The nanoLC set consisting of the EASY-nLC II nanoflow HPLC system (Bruker Daltonics, Bremen, Germany) and the Proteineer-fc II fraction collection device (Bruker Daltonics, Bremen, Germany) was controlled with HyStar 3.2 software (Bruker Daltonics, Bremen, Germany). The nanoLC system parts were the NS-MP-10 BioSphere C18 trap column for protein and peptide concentration (20 mm length, 100 μm inner diameter, pore size 120 Å, particle size 5 μm) (NanoSeparations, Nieuwkoop, The Netherlands) and the Thermo Scientific Acclaim PepMap 100 column (150 mm length, 75 μm inner diameter, pore size 100 Å, particle size 3 μm) (Thermo Scientific, Sunnyvale, CA, USA) for separation. The flow rate for the separation was 300 nL/min, and the linear gradient was 2–50% of ACN for 96 min (mobile phase A: 0.1% TFA in water, mobile phase B: 0.1% TFA in ACN). Then, 22 min before the start of the gradient, 80 µL of each of the 384 nanoLC fractions was mixed with 420 µL of HCCA matrix solution (36 µL of saturated HCCA solution in 0.1% TFA and acetonitrile (ratio 90:10, *v*/*v*), 748 μL of acetonitrile and 0.1% TFA (95:5 ratio, *v*/*v*) mixture, 8 μL of 10% TFA and 8 μL of 100 mM ammonium phosphate) and automatically spotted onto the AnchorChip Standard 800 μm target plate (Bruker Daltonics, Bremen, Germany). The MALDI-TOF/TOF instrument (UltrafleXtreme, Bruker Daltonics, Bremen, Germany) operating in the reflector mode in the mass range *m*/*z* 700–3500 was used for the MS analysis. A total of 4000 spectra were collected per spot. External calibration was performed using a mixture of Peptide Calibration Standard (Bruker Daltonics, Bremen, Germany) with an average mass deviation of less than 1 ppm. WARP-LC software (Bruker Daltonics, Bremen, Germany) was used to establish a list of precursor ions for identification. Appointed *m*/*z* were analysed using the MS/MS mode. Settings for MS and MS/MS mode were as follows: ion source 1, 7.50 kV; ion source 2, 6.75 kV; reflectron 1, 29.50 kV; reflectron 2, 14.00 kV; lens, 3.50 kV; lift 1, 19.00 kV; lift 2, 3.00 kV; pulsed ion extraction time, 80 ns. FlexControl 3.4, FlexAnalysis 3.4 and BioTools 3.2 software (Bruker Daltonics, Bremen, Germany) were used for spectra acquisition, data processing and evaluation. The SwissProt database and Mascot 2.4.1 search engine with taxonomic restriction to Homo sapiens (humans) were used to identify discriminatory proteomic features. The following protein search parameters were used: precursor ion mass tolerance ± 50 ppm; fragmentation mass tolerance *m*/*z* ± 0.7; no enzyme; monoisotopic mass; peptide charge + 1.

### 4.6. Data Analysis

To calculate the allergen frequency, mean sIgE concentration, median and standard deviation, Statistica 13.0 (StatSoft Inc., Tulsa, OK, USA) and MedCalc statistical Software (MedCalc Software Ltd., Ostend, Belgium) were used.

Processing of the obtained MS data was performed using ClinProTools 3.0 chemometric software (Bruker Daltonics, Bremen, Germany). In order for the software to be able to group all replicates of the analysed samples into one biological replica, the spectra grouping function was used. Spectra processing consists of recalibration using the prominent common *m*/*z* values, normalization to the total ion current (TIC), smoothing, the signal-to-noise ratio ≥ 5, baseline top hat subtraction (minimum baseline width: 10%), peak calculation and peak picking procedure. In order to improve the signal-to-noise ratio during the peak picking operation, the total mean spectrum was calculated. Spectra were smoothed and processed in the mass range of 1000–10,000 Da. A comparison of atopic children allergic to storage proteins with the control group was carried out using the Wilcoxon test. Statistical significance was obtained when the *p*-value was ≤0.05. An assessment of the discrimination ability of each peak was obtained by calculating the receiver operating characteristic (ROC) curve and the area under the ROC curve (AUC). Three mathematical algorithms, namely genetic algorithm (GA), supervised neural network (SNN) and quick classifier (QC), were used for the model analysis and the selection of peptide/protein peak clusters. Each model showed a combination of differentiating peaks. A detailed description of all chemometric algorithms is included in our previous publication [48]. For each algorithm, we calculated cross-validation, recognition capability and external validation to determine the reliability of the models. Due to the number of samples, “Leave One Out” mode was used to calculate cross-validation.

## 5. Conclusions

Our results show that the IgE-mediated allergy to plant storage proteins (2S albumins, 7S globulins, 11S globulins and cereal prolamins) is a significant problem in atopic children aged 0–5 years. We clearly emphasized that especially in this group of patients, it is important to distinguish primary food allergies from pollen-associated food allergies. Identification of peptides (cell cycle control protein 50A, testis-expressed sequence 13B, DENN domain-containing protein 5A and SKI family transcriptional corepressor 2) with discriminatory power between patients allergic to storage proteins and other allergy sufferers and healthy individuals is the next crucial step for understanding the molecular consequences of allergy to storage proteins. In the future, MALDI-TOF MS analysis should be complemented with a quantitative approach in a larger set of samples to confirm the results of protein–peptide profiling.

## Figures and Tables

**Table 1:** Results of Allergy Explorer ALEX2.

Superfamily	Family	Allergen	Allergen Source	Concentration (kUA/L)					
				Population			Allergy Group		
				Mean	SD	Median	Mean	SD	Median
Cupins	Vicilins/7S globulins	nAra h 1	Peanut	2.104	7.669	0.1	10.93	14.959	3.165
		nCor a 11	Hazelnut	0.210	0.413	0.1	1.592	0.727	1.6
		nJug r 6	Walnut	0.146	0.189	0.1	0.838	0.399	0.705
		nJug r 2	Walnut	0.187	0.474	0.1	2.223	1.169	2.43
		nPis v 3	Pistachio	0.106	0.039	0.1	0.44	0.0	0.44
		rGly m 5	Soy	-	-	-	-	-	-
	Legumins/11S globulins	nAra h 3	Peanut	1.160	4.820	0.1	9.038	11.216	3.59
		nCor a 9	Hazelnut	0.342	1.113	0.1	3.157	2.662	2.695
		nJug r 4	Walnut	0.206	0.516	0.1	1.662	1.332	1.47
		rGly m 6	Soy	0.391	1.652	0.1	5.623	4.792	4.455
		rAna o 2	Cashew nut	-	-	-	-	-	-
		nPis v 2	Pistachio	-	-	-	-	-	-
Prolamins	2S albumins	nAra h 2	Peanut	2.553	8.335	0.1	14.434	15.359	9.81
		nAra h 6	Peanut	2.376	8.052	0.1	14.513	15.353	7.975
		nCor a 14	Hazelnut	0.315	0.926	0.1	2.767	2.081	2.21
		nSes i 1	Sesame	0.202	0.443	0.1	1.56	0.997	1.19
		nSin a 1	Mustard	0.298	1.193	0.1	5.047	3.549	3.65
		nJug r 1	Walnut	0.425	2.514	0.1	8.287	9.787	2.38
		rAna o 3	Cashew nut	0.141	0.283	0.1	1.127	1.011	0.53
		nMac i	Macadamia	0.149	0.395	0.1	1.94	1.62	1.94
		nBer e 1	Brazil nut	0.107	0.062	0.1	0.64	0.0	0.64
		rPis v 1	Pistachio	0.121	0.179	0.1	1.67	0.0	1.67
		nPap s	Poppy seeds	0.107	0.062	0.1	0.64	0.0	0.64
		nFag e 2	Buckwheat	-	-	-	-	-	-
		rGly m 8	Soy	-	-	-	-	-	-
	Cereal prolamins	rTri a 19 (gliadin)	Wheat	0.113	0.037	0.1	0.3	0.0	0.3

**Table 2:** Peaks (*m*/*z*) discriminating study groups calculated by genetic algorithm (GA), supervised neural network (SNN) and quick classifier (QC).

GA	QC	SNN
1077.88 1219.27 1419.92 1897.85 2661.39 3159.56 3198.01 3689.35 4287.68 4396.62 4645.8 4964.58 7922.04 8126.66 8866.89	1277.39 1773.08 1795.39 1945.54 2012.59 2016.56 2144.89 2269.28 2474.89 2601.66 2726.71 3689.35 4964.58 5004.83 5017.61 5044.33 6065.66 8866.89	1450.43 1795.39 4151.16 5352.47 5905.01 6221.79 8601.73 9135.64

**Table 3:** Chemometric parameters for genetic algorithm (GA), quick classifier (QC) and supervised neural network (SNN).

	GA	QC	SNN
Cross-validation (%)	94.91	67.56	62.29
Recognition capability (%)	99.25	68.61	56.66
Correct classified (%):			
🗸 Allergy	42.7	54.5	0
🗸 Control	94.4	81.5	96.3

**Table 4:** Identified proteins differentiating the study groups.

Precursor Ion *m*/*z*	*p*-Value	UniProtKB-ID	Peptide Sequence	Protein Name
1077.88	0.0158	CC50A_HUMAN	R.NSSNTADITI.-	Cell cycle control protein 50A
1419.92	0.0139	TX13B_HUMAN	K.EACTWGSLALGVR.F	Testis-expressed sequence 13B
2012.59	0.00467	DEN5A_HUMAN	K.LNTGQIQESIGEAVNGIVK.H	DENN domain-containing protein 5A
2601.66	0.00194	SKOR2_HUMAN	K.AGGGSYHHSSAFRPVGGKDDAESLAK.L	SKI family transcriptional corepressor 2
2661.39	0.532	SPAG6_HUMAN	R.LPGIMMLGYVAAHSENLAMAVIISK.G	Sperm-associated antigen 6

**Table 5:** Comparison of major peanut allergen Ara h 1, Ara 2 and Ara h 3 belonging to different protein families (https://www.uniprot.org/ (accessed on 23 December 2022)).

Allergen Characteristics	Ara h 1	Ara h 2	Ara h 3
Source	peanut	peanut	peanut
Protein family	7S globulins	2S albumins	11S globulins
% allergy suffers in the study group	18.4%	17.1%	11.8%
Biological function	seed storage protein	seed storage protein	seed storage protein
Molecular structure	2 β-barrels surrounded by α-helical and unstructured loops 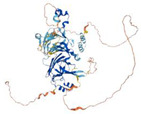 monomer 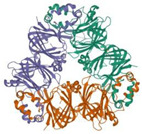 trimer	5 helix bundle held together by 4 disulphide bonds 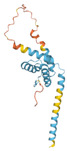 monomer 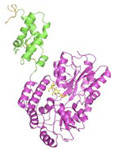 maltose-binding protein (MBP)-Ara h 2 fusion system	2 β-barrels surrounded by α-helical and unstructured loops, composed of 2 disulphide-linked chains; each subunit is composed of an acidic and a basic chain derived from a single precursor and linked by a disulphide bond 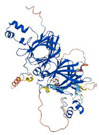 monomer 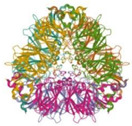 hexamer
Molecular weight	64 kDa	17 kDa	60 kDa, 37 kDa (fragment)
Length	626 aa	157 aa	512 aa
Ligand binding	Small molecular ligands, e.g., Ca^2+^	No	Metal ions, e.g., Mg^2+^
Oligomerization	Trimers	No	Hexamers
Glycosylation	Yes	No	No
Disulphide bonds	No	4	2
Isoelectric point	4.55	5.2	5.5
Heat stability	Yes	Yes	Yes
Denaturation temperature	88.3 °C	110 °C	95 °C

**Table 6:** Characteristics of the subjects.

Characteristics of the Participants	
No. of subjects	76
Sex	
Male	44 (57.9%)
Female	32 (42.1%)
Age (months)	
Median	19
Mean	23, 24
Range	2–60
Eczema (for the past 6 weeks or more)	76
Patient age on the onset of eczema (months)	
Median	3
Mean	5, 12
Range	1–36
Atopic dermatitis (L20)	76
Association with foods:	
Milk	51
Egg	16
Egg white	2
Egg yolk	1
Cocoa	8
Chocolate	2
Oatmeal	1
Flour	1
Wheat	5
Gluten	4
Rye bread	1
Ketchup	1
Sweets	1
Nuts	15
Coconut	2
Fruits	15
Banana	2
Strawberry	5
Apple	3
Peach	1
Citrus	3
Juice	2
Carrot	2
Tomato	1
Potato	1
Soy	1
Chickpeas	1
Silage	1
Allergic Urticaria (L50)	10
Angioedema (T78.3)	5
The causative food:	
Hazelnut	1
Milk	2
Egg	3
Egg white	1
Peanut	2
White fish	1
Raisins	1
Gluten	1
Cauliflower	1
Anaphylactic shock (T78.0)	5
Symptoms of a generalized reaction:	
Dyspnoea	5
Vascular oedema	2
Hives	1
Adrenaline	0
The causative food:	
Hazelnut	1
Milk	1
Peanut	1
Egg	1
Fish (cod)	1
Chronic symptoms of the digestive system:	
Colic	6
Abdominal pain	5
Abdominal gas	3
Vomiting, Downpouring	3
Diarrhoea	6
Constipation	3
Mucus in the stool	2
Blood in the stool	0
Chronic diseases:	
Early childhood asthma	8
Recurrent bronchitis	0
Allergic rhinitis	20
Recurrent upper respiratory tract infections	1
Neurogenic bladder	1

## Data Availability

The data presented in this study are available in Supplementary Files Spreadsheet S1 and Spreadsheet S2.

## Supplementary Materials

The following supporting information can be downloaded at: https://www.mdpi.com/article/10.3390/ijms24021804/s1.
